# ITAlian partnership for psychosis prevention (ITAPP): Improving the mental health of young people

**DOI:** 10.1192/j.eurpsy.2021.2232

**Published:** 2021-09-21

**Authors:** Paolo Fusar-Poli, Amedeo Minichino, Paolo Brambilla, Andrea Raballo, Alessandro Bertolino, Renato Borgatti, Martina Mensi, Adele Ferro, Silvana Galderisi

**Affiliations:** 1 Early Psychosis: Interventions and Clinical-Detection (EPIC) Lab, Department of Psychosis Studies, Institute of Psychiatry, Psychology & Neuroscience, King’s College London, London, United Kingdom; 2 OASIS Service, South London and Maudsley NHS Foundation Trust, London, United Kingdom; 3 Department of Brain and Behavioral Sciences, University of Pavia, Pavia, Italy; 4 National Institute for Health Research, Maudsley Biomedical Research Centre, South London and Maudsley NHS Foundation Trust, London, United Kingdom; 5 Dipartimento di Fisiopatologia Medico-Chirugica e dei Trapianiti, Università degli Studi di Milano La Statale, Milan, Italy; 6 Department of Neurosciences and Mental Health, Fondazione IRCCS Ca’ Granda Ospedale Maggiore Policlinico, Milano, Italy; 7 Dipartimento di Medicina e Chirurgia, Università degli Studi di Perugia, Perugia, Italy; 8 Dipartimento di Scienze Mediche di Base, Neuroscienze e Organi di Senso, Università degli Studi di Bari Aldo Moro, Bari, Italy; 9 Child Neuropsychiatry Unit, IRCCS Mondino Foundation, Pavia, Italy; 10 Dipartimento di Salute Mentale e Fisica e Medicina Preventiva, Università degli Studi della Campania Luigi Vanvitelli, Naples, Italy

**Keywords:** Clinical high risk, early intervention, prevention, psychosis, schizophrenia

## Abstract

**Background:**

The European impact of the clinical high risk for psychosis (CHR-P) paradigm is constrained by the lack of critical mass (detection) to power prognostic and preventive interventions.

**Methods:**

An ITAlian partnership for psychosis prevention (ITAPP) was created across CHR-P centers, which were surveyed to describe: (a) service, catchment area, and outreach; (b) service users; and (c) interventions and outcomes. Descriptive statistics and Kaplan–Meier failure function complemented the analyses.

**Results:**

The ITAPP included five CHR-P clinical academic centers established from 2007 to 2018, serving about 13 million inhabitants, with a recruitment capacity of 277 CHR-P individuals (mean age: 18.7 years, SD: 4.8, range: 12–39 years; 53.1% females; 85.7% meeting attenuated psychotic symptoms; 85.8% without any substance abuse). All centers were multidisciplinary and included adolescents and young adults (transitional) primarily recruited through healthcare services. The comprehensive assessment of at-risk mental state was the most widely used instrument, while the duration of follow-up, type of outreach, and preventive interventions were heterogeneous. Across 205 CHR-P individuals with follow up (663.7 days ± 551.7), the cumulative risk of psychosis increased from 8.7% (95% CI 5.3–14.1) at 1 year to 15.9% (95% CI 10.6–23.3) at 2 years, 21.8% (95% CI 14.9–31.3) at 3 years, 34.8% (95% CI 24.5–47.9) at 4 years, and 51.9% (95% CI 36.3–69.6) at 5 years.

**Conclusions:**

The ITAPP is one of the few CHR-P clinical research partnerships in Europe for fostering detection, prognosis, and preventive care, as well as for translating research innovations into practice.

## Introduction

Indicated prevention implemented in specialized clinical high risk for psychosis (CHR-P) [1] services [2][3][4]has the potential to ameliorate presenting symptoms, delay or prevent the onset of psychosis, and reduce healthcare access and duration of untreated psychosis (secondary prevention) [5,6]. Individuals who meet CHR-P criteria are young help-seeking people (14–35 years old, mean age 21 years [7]) presenting with several risk factors for psychotic disorders [8–10]. CHR-P criteria are assessed with validated semistructured psychometric instruments, which deliver a group-level estimate (i.e., at risk vs. not at risk) [11] robustly associated with psychosis onset (meta-analytic odds ratio = 9.32) [8]. This association is tightly linked to the help-seeking behavior, because the same psychometric tools do not have prognostic value in general population [12]. Other than psychotic onset, clinical trajectories for CHR-P include functional decline [13], persistence of attenuated psychotic symptoms [14], or other psychiatric comorbidities [9].

CHR-P individuals are challenging to detect and follow up. Detection of most individuals at-risk for psychosis is one of the main barriers toward the implementation of the CHR-P paradigm in real-word clinical scenarios [15].

Furthermore, meta-analytic evidence shows that only about 20% of CHR-P individuals develop a psychotic disorder after 2 years from ascertainment [15]. It is therefore pivotal for CHR-P studies to use large samples and adequate follow-up in order to reach adequate statistical power. Considering the poor real-world outcomes associated with psychotic onset [16,17], this is an upmost clinical and research priority. The consequences of inadequate statistical power in CHR-P studies can be directly observed on findings from interventional studies. For instance, while indicated prevention implemented in CHR-P services (e.g., cognitive behavioral therapy [CBT] as for [18]) has the potential to improve outcomes, the largest randomized controlled trials and the most recent network/Cochrane meta-analyses [19,20] on CBT for psychosis prevention held negative results. A recent umbrella review (a review of meta-analyses) indicated that these trials were likely underpowered, because several hundreds of CHR-P individuals would be required to reach critical mass in interventional preventive studies [21].

Implementing large-scale collaborations that focuses on specialized CHR-P clinical services [22] is the mainstream approach to overcome these barriers and advance clinical research to foster translational innovations in this field [7]. Successful examples come from large-scale initiatives, such as the Australian and North American CHR-P consortia [23], which are currently missing in Italy.

The primary aim of the current study is to fill this gap by presenting an innovative ITAlian partnership for psychosis prevention (ITAPP). The core findings will be discussed to provide directions to advance and implement clinical research collaborations in the CHR-P field in Europe, and therefore advance knowledge for the potential benefit of many young people at risk of developing this disorder.

## Methods

This manuscript originates from a workshop on CHR-P services in Italy, held on two occasions (October 2, 2019: Italian Society of Biological Psychiatry; February 21, 2020: Società Italiana di Psicopatologia). Leading clinical and academic centers for psychosis prevention in Italy (Pavia, Milan, Perugia, Bari, and Naples) attended the workshop. We leveraged an empirical framework (internationally validated by our team [22]) for describing core characteristics of CHR-P services (Table 1): (a) type of CHR-P service (i.e., standalone vs. integrated model), catchment area, and pathways to care (i.e., outreach strategies and sources of referral); (b) service users; and (c) interventions and outcomes (i.e., psychosis onset, functioning, and persistence/remission of symptoms). For standalone CHR-P services, we defined a separate team that works independently from other generic community mental health teams. For integrated CHR-P services, we defined a team wholly embedded into community mental healthcare services, with professionals that adopt principles of early intervention in psychosis [24].

**Table 1.** Characteristics of the ITAPP sites.

CHR-P site	Type of service^a^	Catchment area	Pathways to care	Interventions and outcomes
Pavia	Standalone (CHR-P < 18 y.o.)	~550,000 inhabitants	*Referral sources*	*Interventions*
	Integrated (CHR-P > 18 y.o.)		1. Secondary mental health services	1. Integrated treatments (medications + psychotherapy) targeting CHR-P comorbidities
			2. General practitioners	*Outcomes*
			3. Pediatricians	1. Psychotic onset, remission of CHR-P criteria, functioning, and cognition
			4. Self-referrals	
			*Outreach strategies*	
			1. Digital outreach	
Bari	Integrated	~750,000 inhabitants	*Referral sources*	*Interventions*
			1. Secondary mental health services	1. Integrated treatments (medications + psychotherapy) targeting CHR-P comorbidities
			2. General practitioners	*Outcomes*
			3. Self-referrals	1. Psychotic onset, remission of CHR-P criteria, functioning, and cognition
			*Outreach strategies*	
			1. Public engagement/Screening in schools	
Naples	Integrated	~1,000,000 inhabitants	*Referral sources*	*Interventions*
			1. Secondary mental health services	1. Integrated treatments (medications + psychotherapy) targeting CHR-P comorbidities
			2. General practitioners	*Outcomes*
			3. Self-referrals	1. Psychotic onset, remission of CHR-P criteria, functioning, and cognition
Perugia	Standalone	~900,000 inhabitants	*Referral sources*	*Interventions*
			1. Secondary mental health services	1. Recommendations to referral sources (does not provide direct treatment)
			2. General practitioners	*Outcomes*
			3. Pediatricians	1. Psychotic onset and remission of CHR-P
Milan	Integrated	~250,000 inhabitants	*Referral sources*	*Interventions*
			1. Secondary mental health services	1. Integrated treatments (medications + psychotherapy) targeting CHR-P comorbidities
			2. General practitioners	*Outcomes*
			3. Self-referrals	1. Psychotic onset, remission of CHR-P criteria, functioning, and cognition

*Abbreviations:* CHR-P, clinical high risk for psychosis; ITAPP, ITAlian partnership for psychosis prevention.

a Standalone or integrated CHR-P services. For standalone CHR-P services, we defined a separate team that works independently from other generic community mental health teams. For integrated CHR-P services, we defined a team wholly embedded into community mental healthcare services, with professionals that adopt principles of early intervention in psychosis.

When available, quantitative data were descriptively summarized using mean (and median) and SD for continuous variables and frequencies for categorical variables. Furthermore, for each CHR-P service, we collected the baseline sample size, the individual follow-up time, and the number of events (transition to psychosis from a CHR-P stage). The ascertainment of the CHR-P state and psychotic onset were determined through standardized psychometric instruments, including the comprehensive assessment of at-risk mental state (CAARMS) [25]; the structured interview for prodromal syndrome (SIPS) [26]; the schizophrenia proneness instrument, adult version (SPI-A) [27]; and the schizophrenia proneness instrument, child and youth version (SPI-CY) [27], in line with international clinical practice in this field [7]. Assessment of substance misuse was based on local healthcare guidelines (mainly laboratory diagnostic and anamnesis). Substance misuse was not an exclusion criterion during assessment procedures. The cumulative incidence risk of transition to psychosis during the follow-up period was estimated with Kaplan–Meier survival analysis. These data were then plotted and analyzed with a failure function (1-Kaplan–Meier) across the overall ITAPP sample, and point estimates were reported for each year. A failure function was also used to illustrate the pattern of transition risk across ITAPP centers. The Kaplan–Meier was truncated when less than 10 CHR-P individuals were still at risk. Analyses were performed using STATA16, and *p*-value was set to 0.05 two-sided.

## Results

### CHR-P center in Pavia

#### Service, catchment area, and pathways to care

The CHR-P center in Pavia encompasses two main sites: a child and adolescent neuropsychiatric unit and an outpatient clinic for young adults.

The child and adolescent neuropsychiatry unit at the IRCCS Istituto Neurologico Casimiro Mondino was set up in 2007. This unit offers assessment and preventive care to help-seeking children and adolescents under the age of 18 years as part of an integrated team adopting principles of early intervention in the context of existing mental health services. The outpatient clinic is a standalone CHR-P service set up in 2019 at the Department of Brain and Behavioral Sciences. This service targets young adults (aged 18–35) with potentially emerging severe mental disorders (including bipolar and depression) and works closely with local adult psychiatric mental health services.

The research activities of these two units are embedded with those of the University of Pavia. The clinical team includes psychiatrists, psychologists, nurses, trainees, and social workers. The catchment area extends from Pavia and its surroundings (545,888 inhabitants) to the Lombardy region (10,060,000 inhabitants), with additional CHR-P referrals routinely received from all over Italy.

The primary referral sources for CHR-P are pediatricians, general practitioners, other child and adolescent mental health services in the community, and self-referrals. This center is implementing a digital outreach for CHR-P.

#### Service users

This center treated 129 CHR-P individuals over the past years. Being mainly embedded in child and adolescent mental health services, CHR-P mean age and age range in this site are lower than those observed in the other Italian sites (Table 2). The assessment is based on the CAARMS. The large majority (98.4%) of CHR-P individuals met criteria for attenuated psychosis symptoms (APSs), and a minority had ever used substances (18.7%; see also Table 2). CHR-P criteria were often comorbid with other psychiatric diagnoses, the most prevalent ones being personality disorders (36.8%), major depressive disorders (27.5%), and generalized anxiety disorders (15%).

**Table 2.** Baseline sociodemographic and clinical characteristics of CHR-P individuals in the ITAPP.

	Total ITAPP sample	Pavia	Bari	Naples	Perugia	Milan	Test	*p*
Sample size (*n*)	277	129	72	36	24	16		
Age (years [mean ± SD])	18.7 ± 4.8	15.4 ± 1.5	20.9 ± 4.6	23.4 ± 5.1	18.2 ± 2.9	24.6 ± 6.0	*F* = 43.5	<0.001
Age (years [range])	12–39	12–18	15–35	16–39	15–28	16–39		
Gender								
Females (%)	53.1	60.2	47.2	44.4	45.8	47.1	*X* ^2^ = 2.8	0.6
Males (%)	46.9	39.8	52.8	55.6	54.2	52.9		
Any substance misuse								
Yes (%)	14.2	3.1	18.7	19.4	45.4	31.3	*X* ^2^ = 21.8	<0.001
No (%)	85.8	96.9	81.3	80.6	54.6	68.7		
CHR-P assessment								
Psychometric instrument		CAARMS	CAARMS, SIPS, and SPI-A	CAARMS and SPI-A	CAARMS	CAARMS, SIPS, and SPI-A/SPI-CY	–	–
CHR-P subgroup^a^								
BLIPSs (%)	6.1	0	6.9	19.4	36.4	0	–	–
APSs (%)	85.7	98.4	73.6	86.1	27.3	75.0	–	–
GRD (%)	19.5	4.6	20.8	13.9	100	31.2	–	–

*Abbreviations:* APS, attenuated psychotic symptom; BLIPS, brief limited intermittent psychotic symptom; CAARMS, comprehensive assessment of the at-risk mental state; CHR-P, clinical high risk for psychosis; GRD, genetic risk and deterioration syndrome; ITAPP, ITAlian partnership for psychosis prevention; SIPS, structured interview for prodromal symptoms; SPI-A, schizophrenia proneness instrument, adult version; SPI-CY, schizophrenia proneness instrument, child and youth version.

a Subgroups may overlap, so the total count is not 100%.

#### Interventions and outcomes

CHR-P individuals are actively followed up over time. A yearly routine CAARMS assessment is offered to all individuals, with contingent assessments upon eventual clinical deterioration. The main outcomes of interest are psychotic onset, functioning (global, role, and social), cognition, persistence/remission of CHR-P criteria, and mental health comorbidities.

This center provides integrated interventions (medications alone, medications + psychotherapy, psychotherapy alone, and psychosocial support), which are mainly aimed at improving the mental health of young CHR-P individuals and their comorbidities.

### CHR-P center in Bari

#### Service, catchment area, and outreach

The Individuazione Precoce del Rischio Psicotico(IPRR) CHR-P center is a section of a general adult mental health service. It was set up in 2015 to offer assessment and preventive care to help-seeking young adults (aged 18 and above). The is an integrated service that adopts principles of early intervention in the context of an outpatient clinic dedicated to the treatment of psychotic disorders. The research activities of are embedded with those of the University of Bari. The clinical team includes psychiatrists, psychologists, and social workers. The catchment area includes Bari metropolitan area (more than 750,000 inhabitants) and further extends to the whole region of Puglia, Italy (more than 4 million inhabitants).

The main referral sources are local mental health services and general practitioners. The also provides outreach activities to improve pathways to care for CHR-P such as public engagement and screening activities in school and scheduled outreach sessions in local mental health services.

#### Service users

The treated 72 CHR-P individuals over the past years. This unit employs the CAARMS, the SIPS, and the SPI-A. In this site, the large majority (73.6%) of CHR-P individuals met the APS criteria, and a small minority had ever used substances (14.2%; see also Table 2). CHR-P criteria were often comorbid with other psychiatric diagnoses, but these comorbid diagnoses are not systematically recorded.

#### Interventions and outcomes

The mainly offers psychotherapy interventions and medications (at minimal effective dose) when required. The main outcomes of interest are psychotic onset, functioning, cognition, persistence/remission of CHR-P criteria, and mental health comorbidities.

### CHR-P center in Naples

#### Service, catchment area, and outreach

The CHR-P center in Naples is a section of a general adult mental health service. It was set up in 2015 to offer assessment and preventive care to help-seeking young adults (aged 18 and above). This CHR-P center is an integrated service that adopts principles of early intervention in the context of an outpatient clinic dedicated to the treatment of psychotic illness. The research activities of this center are embedded with those of the University of Campania Vanvitelli. The team includes academic personnel (clinical academics and PhD students), psychiatrists (consultants and trainees), psychologists, nurses, and social workers.

The catchment area consists of an established network of 13 mental health departments located in the city of Naples and across the Campania region (from 164,000 to 1,000,000 inhabitants). The main referral sources are mental health professionals, emergency departments, liaison psychiatry, general practitioners, and self-referrals.

#### Service users

The Vanvitelli outpatient unit for psychotic disorders screens about 500 patients every year. Approximately, 5% of these patients are CHR-P. This center treated 36 CHR-P individuals over the past years.

This unit uses the CAARMS and the SPI-A. The large majority (86.1%) of CHR-P individuals met the APS criteria, and a minority had ever used substances (19.4%; see also Table 2). More than half CHR-P individuals (52.8%) had psychiatric comorbidity, the most prevalent being generalized anxiety disorder and panic disorder.

#### Interventions and outcomes

Most CHR-P individuals are offered psychotherapy, cognitive remediation, and psychoeducation. Medications are also frequently used, most often in combination with psychotherapy. The use of medications is mainly intended to treat mental health comorbidities. The main outcomes of interest are psychotic onset, functioning, cognition, persistence/remission of CHR-P criteria, and mental health comorbidities. CHR-P individuals are followed up over time with clinical interviews to identify outcomes of interest. There is no defined follow-up duration. Up to now, the longest follow-up has been 58 months (range: 2–58; mean: 17).

### CHR-P center in Perugia

#### Service, catchment area, and outreach

The Centre for Translational, Phenomenological and Developmental Psychopathology (CTPDP) is an outpatient unit, which was set up in 2018. The recruitment targets of this center are children, adolescents, and young adults (age range: 11–25 years), and it consists of a standalone second-level outpatient unit performing early differential diagnosis, monitoring and treatment supervision advice in synergy with the network of local services across Umbria Region. The research activities of the CTPDP are embedded with those of the University of Perugia. Due to the circumscribed staffing of the clinical team, which includes an academic psychiatrist (25% of time), two residents (20% of time), and two volunteer psychologists in rotational training (25% of time), integrated community treatment is performed through the referring network of local MH services following consolidated local practices and habits. The catchment area of the CTPDP is the Italian region of Umbria (about 900,000 inhabitants). The CTPDP has a pan-regional outreach receiving referrals from the two local National Health Service Trusts. The referral sources are local mental health services across the Umbria region which also scrutinize potential referrals from general practitioners and pediatricians based on clinical need of care.

#### Service users

The CTPDP included 24 CHR-P individuals (assessed with the CAARMS). About one-third of CHR-P met criteria for brief limited intermittent psychotic symptoms (36.4%) and a substantial proportion of the sample met criteria for genetic risk and deterioration syndrome (see also Table 2). A minority of CHR-P presented other psychiatric comorbidities; these included specific learning disabilities, and obsessive–compulsive disorders.

#### Interventions and outcomes

The also offers to the referring MH services the opportunity for regular longitudinal follow-up on a semester/annual basis, with the outcome of interests being psychotic onset, hospitalization, and symptoms trajectories.

The CTPDP does not offer direct preventive care to CHR-P, but it provides comprehensive recommendations for treatment to the referring service.

### CHR-P center in Milan

#### Service, catchment area, and outreach

The CHR-P center in Milan is a section of a general adult mental health service located within the Department of Neurosciences and Mental Health of the IRCCS Ca’ Granda Ospedale Maggiore Policlinico. It was set up in 2016 to treat help-seeking adults (aged 18 and above). It is an integrated service that adopts the principles of early intervention within a larger outpatient unit for general adult psychiatry. The clinical team includes psychiatrists, psychologists, nurses, and social workers. The catchment area includes about 250,000 individuals. The main referral sources are mental health professionals, general practitioners, and self (or family)-referrals.

#### Service users

This center treated 16 CHR-P individuals over the past few years. Standard assessment instruments include the CAARMS, the SIPS, the SPI-A, and the SPI-CY. The large majority (75.0%) of CHR-P individuals met APS criteria, and about one-third had ever used substances (31.3%; see also Table 2). CHR-P criteria were often comorbid with other psychiatric diagnoses, including major depressive disorders, personality disorders, and anxiety disorders.

#### Interventions and outcomes

CHR-P undergo active monitoring with clinical interviews and cognitive testing scheduled every 3 months for up to 18 months. The main outcomes of interest are psychotic onset, functioning, cognition, persistence/remission of CHR-P criteria, and mental health comorbidities.

The CHR-P center in Milan offers multidisciplinary interventions to CHR-P such as medications, psychotherapy, social skills training, cognitive remediation, and physical exercise.

### Baseline sociodemographic and clinical characteristics of the ITAPP

**Figure 1.**
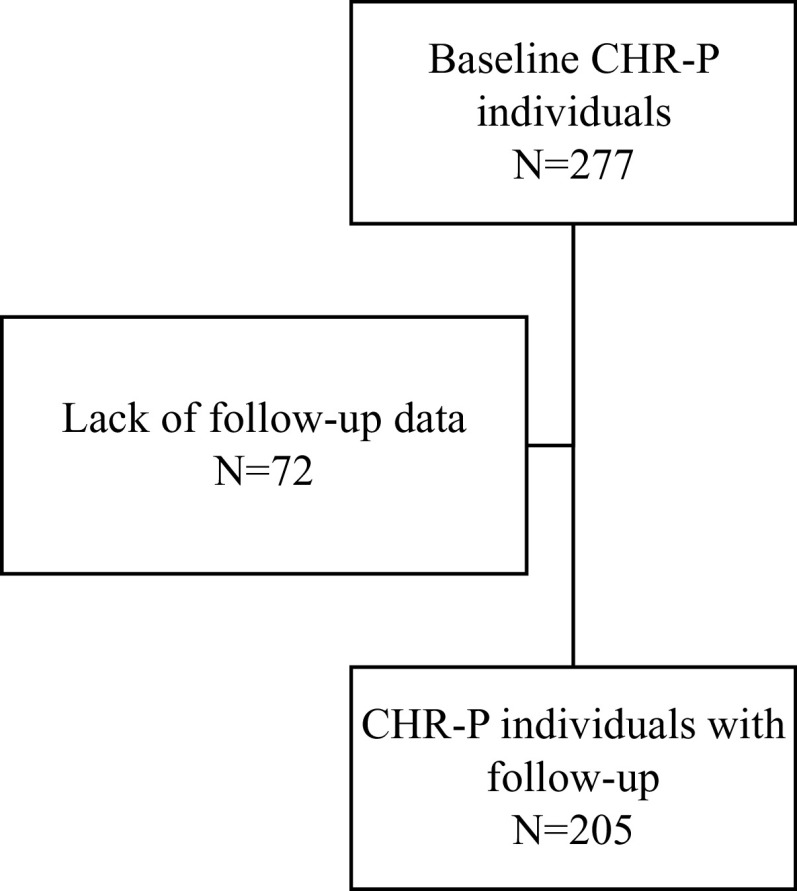
Flowchart of selection of study population.

A total of 277 CHR-P individuals were treated within the ITAPP (129 in Pavia, 72 in Bari, 36 in Naples, 24 in Perugia, and 16 in Milan), for up to 6 years (Figure 1), across a total catchment area of approximately 12,960,000 Italian inhabitants. Overall, CHR-P individuals were aged 18.7 years (SD: 4.8; range: 12–39 years), mostly females (53.1%), without any substance abuse (85.8%); more frequently meeting the APS subgroup (85.7%) of the CHR-P state. The main clinical and sociodemographic characteristics of the baseline sample are reported in Table 2. Main differences between sites were: age, with the youngest CHR-P being in Pavia (15.4 ± 5.1) and the oldest in Milan (24.6 ± 6.0); and rates of substance misuse, with the lowest rates in Pavia (3.1%) and the highest in Perugia (45.4%) and Milan (31.3%).

### Longitudinal risk of psychosis onset in the overall ITAPP sample

A total of 205 CHR-P individuals in the ITAPP had follow-up data (Figure 1). Their mean ± SD follow-up time was 663.7 ± 551.7 days (min: 5; median: 429; max: 2361).

**Figure 2.**
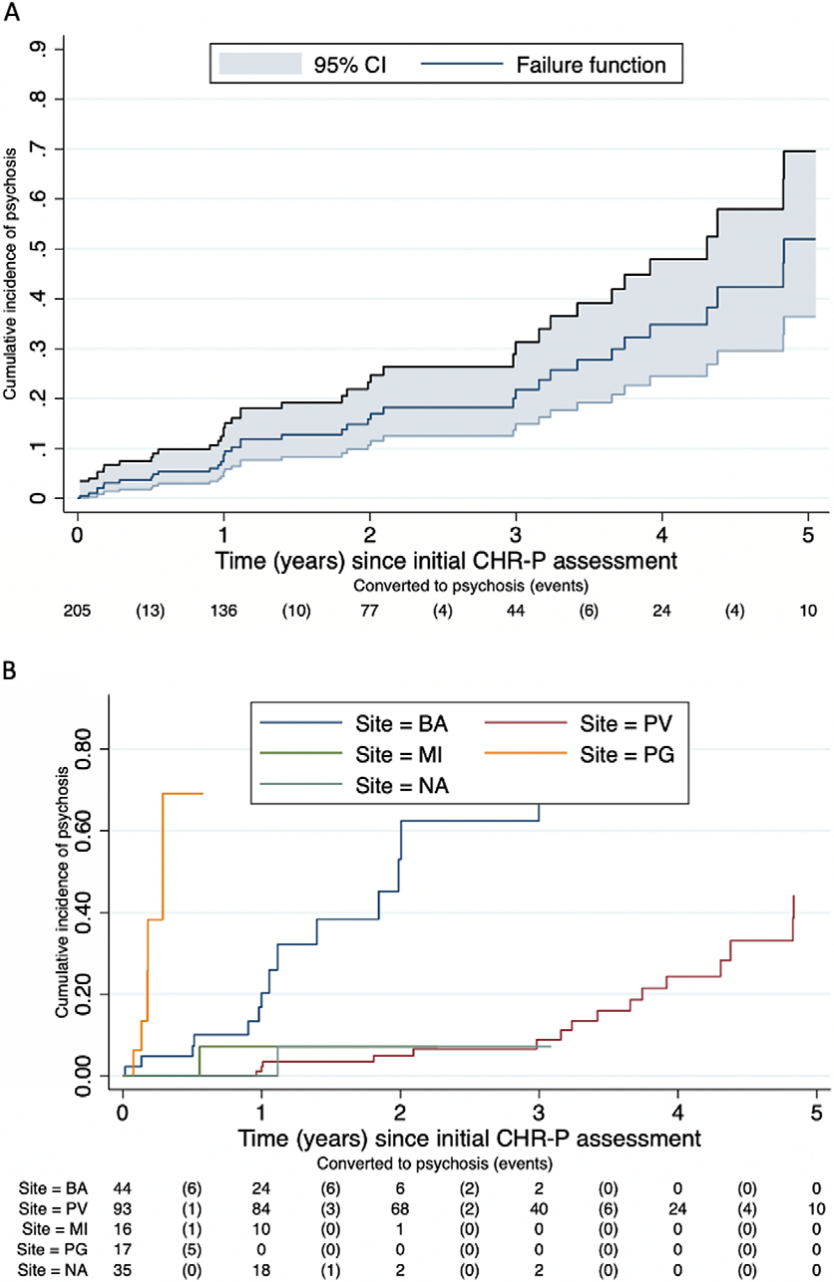
Cumulative incidence of psychosis in the ITAPP. (A) Overall sample. (B) By the ITAPP center. *Abbreviations:* BA, Bari; MI, Milan; NA, Naples; PG, Perugia; PV, Pavi.

Thirty-seven of the 205 CHR-P subjects transitioned to a first episode of psychosis, with a mean ± SD time to conversion of 673.8 ± 551.8 days (min: 6; median: 407; max: 1764). Those nonconverted were followed up for a mean ± SD of 661.5 ± 553.3 days (min: 6; median: 434; max: 1764). Figure 2 presents the cumulative risk of psychosis in the ITAPP. The cumulative risk of psychosis increased from 8.7% (95% CI 5.3–14.1) at 1 year to 15.9% (95% CI 10.6–23.3) at 2 years, 21.8% (95% CI 14.9–31.3) at 3 years, 34.8% (95% CI 24.5–47.9) at 4 years, and 51.9% (95% CI 36.3–69.6) at 5 years (see also Table 3). As highlighted in Figure 2, the main between-site differences were noted in the Perugia site, with most events (transition to psychosis) occurring during the first year of follow-up and in Pavia, where the majority of events occurred in Years 3 and 4. The other three sites (Bari, Naples, and Milan) showed similar failure curves.

**Table 3.** Number of transitions to psychosis and cumulative risk of transition over follow-up in the ITAPP.

Follow-up time	*N* of individuals at CHR-P at risk	*N* of transitions to psychosis	Cumulative risk of transition to psychosis	95% CI	
0.5	170	7	0.036	0.017	0.075
1	136	8	0.087	0.022	0.053
1.5	97	5	0.127	0.083	0.192
2	77	3	0.159	0.106	0.233
2.5	58	2	0.182	0.124	0.263
3	45	2	0.218	0.149	0.313
3.5	36	3	0.278	0.192	0.391
4	25	3	0.348	0.245	0.479
4.5	14	2	0.423	0.296	0.579
5	11	2	0.520	0.363	0.696

*Abbreviations:* CI, confidence interval; *CHR-P, clinical high risk for psychosis; ITAPP, ITAlian partnership for psychosis prevention.*

## Discussion

The ITAPP included five CHR-P centers across Italy, with an overall recruitment capacity of 277 CHR-P individuals. All centers had strong academic links, were multidisciplinary, and provided care to both adolescents and young adults (transitional) mainly recruited through existing healthcare services. The CAARMS was the most widely used assessment instrument, while the duration of follow-up, type of outreach, and provision of preventive interventions were highly heterogeneous. Across 205 CHR-P individuals with follow-up, the cumulative risk of psychosis was 51.9% at 5 years.

The five ITAPP CHR-P centers were set up between 2007 and 2018 across Northern, Central, and Southern Italy, serving a total population of about 13 million inhabitants. All centers were linked to academic departments and conducting research (see below). All ITAPP centers were targeting a transitional age (12–39 years), with minor variations across sites. This is relevant, because a large-scale meta-analysis found that 12.3% of psychotic disorders occur before the age of 18 and 47.8% before 25, with a peak onset at 20.5 years [28]. These findings do not support the current pediatric–adult bifurcation of mental health services (cut at the age of 15 or 18) in Italy, where young people may fall into institutional gaps when they are most liable to mental disorders [29]. By overcoming this limitation, the ITAPP represents a promising template for transitional mental health services [4] aimed at early detection and intervention [3]. In line with these considerations, ITAPP services were enriched with multidisciplinary teams and approaches.

The outreach activity across ITAPP centers was heterogeneous. Most centers relied on secondary mental healthcare and a minority on primary care. Community outreach efforts were present, but limited by challenges such as the dilution of risk-enrichment and the invalidity of CHR-P assessment in the general population [10,12].

CHR-P recruited in ITAPP appeared slightly younger (mean age: 18.7 years) and more frequently females (53.1%) than the average CHR-P population (meta-analytic mean age: 20; 55.28% males) [15]. These differences are likely due to the substantive child and adolescent mental health service component of the ITAPP.

All ITAPP centers employed the CAARMS, with a minority additionally using the SIPS and the SPI-A/SPI-CY. There is no evidence that using different CHR-P instruments impacts the probability of developing psychosis [15]. In line with meta-analytic evidence on CHR-P, most (~85%) CHR-P in the ITAPP presented with attenuated psychotic symptoms [7]. In the light of the relatively young age, most (~85%) of the ITAPP cohort did not present significant substance abuse. These general characteristics are informative in the context of the sampling biases that often affect CHR-P studies: unsystematic detection of CHR-P based on referrals made on suspicion of psychosis risk by heterogenous sources [30]. These idiosyncratic sampling strategies lead to significant risk enrichment (about 15% risk of psychosis onset at 48 months) [30] during the recruitment of young individuals undergoing CHR-P assessment [7].

All ITAPP centers but one provide preventive care: psychotherapy (including psychodynamic approaches), psychosocial support, medications, cognitive remediation, physical exercise, and their combination. Therapies were heterogenous across sites, but they all aimed at treating presenting CHR-P symptoms or their mental comorbidities. While all ITAPP services routinely incorporate comprehensive needs-based interventions, only a few implemented public health initiatives to foster mental health literacy and promote good mental (e.g., resilience and good lifestyle behaviors) and physical health [7]. Such heterogeneity may reflect the lack of robust evidence to favor any specific intervention to improve outcomes of CHR-P individuals and the absence of national clinical guidelines for the prevention of psychosis. Interestingly, most ITAPP centers provided comprehensive monitoring of outcomes encompassing psychotic onset, functioning (global, role, and social), cognition, persistence/remission of CHR-P criteria and symptom trajectories, hospitalization, and mental comorbidities, although the instruments used to measure these outcomes were unstandardized and heterogeneous across sites. The follow-up period was also heterogeneous across sites, with a mean length of about 1.5 years and a maximum of 6 years (Pavia site). The follow-up of our cohort aligned with the average follow-up observed worldwide [22], which is typically not sufficient to capture the long-term outcomes of this patient population [16].

The transition risk to psychosis in ITAPP was at the highest in the first months after the initial CHR-P assessment and it did not plateau until about 4 years, in line with recent meta-analytic analyses in this field, showing that 25% of individuals at CHR-P developed psychosis within 3 years and that transition risk continued increasing in the long term [15]. These findings are also in line with the evidence that the probability of developing other severe real-world outcomes in CHR-P individuals (e.g., risk of hospital admission and increase in psychotropic medications) doubles from the short to the long term [16].

We noticed differences in the cumulative risk of psychosis across different ITAPP sites. These differences might be related to the heterogeneous recruitment and sampling procedures across sites, which resulted in heterogeneous sociodemographic characteristics (i.e., age, substance misuse, and CHR-P subgroup) of CHR-P individuals (Table 2). This heterogeneity further suggests that only large-scale recruitment efforts can provide an accurate representation of real-word scenarios in CHR-P research and provide further strength to the ITAPP initiative.

Conducting long-term follow-ups emerged as core empirical barrier to implementing CHR-P services in the national healthcare system. Additional challenges encompassed difficulties in CHR-P detection [31], the need to reinforce the liaison with schools, universities, general hospitals, and practitioners, the lack of standardized training for staff, and the fragmentation of child/adult and general adult/addiction mental health services. A shared constrain of the ITAPP centers was the lack of dedicated funding and limited human resources to upscale the detection, prognosis, and preventive activity.

Despite these crucial challenges, most ITAPP centers are actively conducting CHR-P research. This level of activity includes implementation studies [32], local cohort studies encompassing psychopathology, neurocognition, neuroimaging to improve prediction of outcomes [33], evidence-based medicine analyses of the CHR-P literature [15], [34][35][36], and prospective studies validating the prognostic accuracy of CHR-P criteria such as the DSM-5 APS [37]. Furthermore, some ITAPP centers have participated in international CHR-P studies, such as PRONIA [17] and PSYSCAN [38], or are founding members of the international clinical research infrastructures, such as the European College of Neuropsychopharmacology Prevention of Mental Disorders and Mental Health Promotion Network [39,40].

The ITAPP initiative has therefore the potential to significantly advance and implement clinical research collaborations in the CHR-P field in Italy, in Europe, and worldwide.

The current data indicate that the ITAPP centers have the capacity to recruit meaningful samples of CHR-P individuals on the national level, to follow them up with established instruments and to offer them potentially preventive interventions. At the same time, the current study highlights important barriers to large-scale implementation of CHR-P prevention such as the lack of a standard data acquisition dataset to record individual participant data and the need of more effective interventions. Future directions include establishing a national CHR-P program using harmonized assessment and follow-up measurements and testing the feasibility of standardized preventative interventions (e.g., good cannabinoids) across the whole ITAPP CHR-P population.

## Conclusions

The ITAPP is one of the few CHR-P clinical research partnerships in Europe for fostering detection, prognosis, and preventive care, as well as for translating research innovations into practice.

## Data Availability

The authors did not give permission to share data.

## References

[1] Fusar-Poli P. The clinical high-risk state for psychosis (CHR-P), version II. Schizophr Bull. 2017;43(1):44–7. doi:10.1093/SCHBUL/SBW158 28053129 PMC5216870

[2] Fusar-Poli P, Estradé A, Spencer TJ, Gupta S, Murguia-Asensio S, Eranti S, et al. Pan-London network for psychosis-prevention (PNP). Front Psychiatry. 2019;10:707. doi:10.3389/FPSYT.2019.00707 31681029 PMC6798006

[3] Kotlicka-Antczak M, Podgórski M, Oliver D, Maric NP, Valmaggia L, Fusar-Poli P. Worldwide implementation of clinical services for the prevention of psychosis: the IEPA early intervention in mental health survey. Early Interv Psychiatry. 2020;14(6):741–50. doi:10.1111/EIP.12950 32067369

[4] Fusar-Poli P, Spencer T, De Micheli A, Curzi V, Nandha S, McGuired P. Outreach and support in South-London (OASIS) 2001–2020: twenty years of early detection, prognosis and preventive care for young people at risk of psychosis. Eur Neuropsychopharmacol. 2020;39:111–22. doi:10.1016/J.EURONEURO.2020.08.002 32921544 PMC7540251

[5] Oliver D, Davies C, Crossland G, Lim S, Gifford G, McGuire P, et al. Can we reduce the duration of untreated psychosis? A systematic review and meta-analysis of controlled interventional studies. Schizophr Bull. 2018;44(6):1362–72. doi:10.1093/SCHBUL/SBX166 29373755 PMC6192469

[6] Fusar-Poli P, McGorry PD, Kane JM. Improving outcomes of first-episode psychosis: an overview. World Psychiatry. 2017;16(3):251–65. doi:10.1002/WPS.20446 28941089 PMC5608829

[7] Fusar-Poli P, de Pablo GS, Correll CU, Meyer-Lindenberg A, Millan MJ, Borgwardt S, et al. Prevention of psychosis: advances in detection, prognosis, and intervention. JAMA Psychiatry. 2020;77(7):755–65. doi:10.1001/JAMAPSYCHIATRY.2019.4779 32159746

[8] Radua J, Ramella-Cravaro V, Ioannidis JPA, Reichenberg A, Phiphopthatsanee N, Amir T, et al. What causes psychosis? An umbrella review of risk and protective factors. World Psychiatry. 2018;17(1):49–66. doi:10.1002/WPS.20490 29352556 PMC5775150

[9] Oliver D, Reilly TJ, Boy OB, Petros N, Davies C, Borgwardt S, et al. What causes the onset of psychosis in individuals at clinical high risk? A meta-analysis of risk and protective factors. Schizophr Bull. 2020;46(1):110–20. doi:10.1093/SCHBUL/SBZ039 31219164 PMC6942149

[10] Fusar-Poli P, Rutigliano G, Stahl D, Schmidt A, Ramella-Cravaro V, Hitesh S, et al. Deconstructing pretest risk enrichment to optimize prediction of psychosis in individuals at clinical high risk. JAMA Psychiatry. 2016;73(12):1260–7. doi:10.1001/JAMAPSYCHIATRY.2016.2707 27784037

[11] Fusar-Poli P, Cappucciati M, Rutigliano G, Schultze-Lutter F, Bonoldi I, Borgwardt S, et al. At risk or not at risk? A meta-analysis of the prognostic accuracy of psychometric interviews for psychosis prediction. World Psychiatry. 2015;14(3):322–32. doi:10.1002/WPS.20250 26407788 PMC4592655

[12] Fusar-Poli P. Why ultra high risk criteria for psychosis prediction do not work well outside clinical samples and what to do about it. World Psychiatry. 2017;16(2):212–3. doi:10.1002/WPS.20405 28498578 PMC5428173

[13] Fusar-Poli P, Rocchetti M, Sardella A, Avila A, Brandizzi M, Caverzasi E, et al. Disorder, not just state of risk: meta-analysis of functioning and quality of life in people at high risk of psychosis. Br J Psychiatry. 2015;207(3):198–206. doi:10.1192/BJP.BP.114.157115 26329563

[14] Fusar-Poli P, Borgwardt S, Bechdolf A, Addington J, Riecher-Rössler A, Schultze-Lutter F, et al. The psychosis high-risk state: a comprehensive state-of-the-art review. JAMA Psychiatry. 2013;70(1):107–20. doi:10.1001/JAMAPSYCHIATRY.2013.269 23165428 PMC4356506

[15] de Pablo GS, Radua J, Pereira J, Bonoldi I, Arienti V, Besana F, et al. Probability of transition to psychosis in individuals at clinical high risk: an updated meta-analysis. JAMA Psychiatry. 2021;78:970–8. doi:10.1001/JAMAPSYCHIATRY.2021.0830 34259821 PMC8281006

[16] Fusar-Poli P, De Micheli A, Signorini L, Baldwin H, de Pablo GS, McGuire P. Real-world long-term outcomes in individuals at clinical risk for psychosis: the case for extending duration of care. EClinicalMedicine. 2020;28:100578. doi:10.1016/J.ECLINM.2020.100578 33294806 PMC7700893

[17] Koutsouleris N, Kambeitz-Ilankovic L, Ruhrmann S, Rosen M, Ruef A, Dwyer DB, et al. Prediction models of functional outcomes for individuals in the clinical high-risk state for psychosis or with recent-onset depression: a multimodal, multisite machine learning analysis. JAMA Psychiatry. 2018;75(11):1156–72. doi:10.1001/JAMAPSYCHIATRY.2018.2165 30267047 PMC6248111

[18] NICE. 1. Recommendations. Psychosis and schizophrenia in adults: prevention and management. Guidance, https://www.nice.org.uk/guidance/cg178/chapter/1-Recommendations#preventing-psychosis-2 [accessed 23 August 2021].

[19] Davies C, Cipriani A, Ioannidis JPA, Radua J, Stahl D, Provenzani U, et al. Lack of evidence to favor specific preventive interventions in psychosis: a network meta-analysis. World Psychiatry. 2018;17(2):196–209. doi:10.1002/WPS.20526 29856551 PMC5980552

[20] Bosnjak Kuharic D, Kekin I, Hew J, Rojnic Kuzman M, Puljak L. Interventions for prodromal stage of psychosis. Cochrane Database Syst Rev. 2019;2019(11):CD012236. doi:10.1002/14651858.CD012236.PUB2 PMC6823626 31689359

[21] Fusar-Poli P, Davies C, Solmi M, Brondino N, De Micheli A, Kotlicka-Antczak M, et al. Preventive treatments for psychosis: umbrella review (just the evidence). Front Psychiatry. 2019;10:764. doi:10.3389/FPSYT.2019.00764 31920732 PMC6917652

[22] de Pablo GS, Estradé A, Cutroni M, Andlauer O, Fusar-Poli P. Establishing a clinical service to prevent psychosis: what, how and when? Systematic review. Transl Psychiatry. 2021;11(1):43. doi:10.1038/S41398-020-01165-X 33441556 PMC7807021

[23] Addington J, Liu L, Brummitt K, Bearden CE, Cadenhead KS, Cornblatt BA, et al. North American Prodrome Longitudinal Study (NAPLS 3): methods and baseline description. Schizophr Res. Published online 18 April 2020. doi:10.1016/J.SCHRES.2020.04.010 PMC7572535 32317224

[24] Schirmbeck F, van der Burg NC, Blankers M, Vermeulen JM, McGuire P, Valmaggia LR, et al. Impact of comorbid affective disorders on longitudinal clinical outcomes in individuals at ultra-high risk for psychosis. Schizophr Bull. Published online 21 August 2021. doi:10.1093/SCHBUL/SBAB088 PMC8781381 34417795

[25] Yung AR, Yuen HP, McGorry PD, Phillips LJ, Kelly D, Dell’Olio M, et al. Mapping the onset of psychosis: the comprehensive assessment of at-risk mental states. Aust N Z J Psychiatry. 2005;39(11–12):964–71. doi:10.1111/J.1440-1614.2005.01714.X 16343296

[26] Pandurangi AK. *The Psychosis-Risk Syndrome: Handbook for Diagnosis and Follow-Up.* By T. H. McGlashan, B. C. Walsh and S. W. Woods. (Pp. 243; $54.95; ISBN 978 0199733316.) USA: Oxford University Press. 2010. Psychol Med. 2011;41(8):1785–6. doi:10.1017/S0033291711000687

[27] Fux L, Walger P, Schimmelmann BG, Schultze-Lutter F. The schizophrenia proneness instrument, child and youth version (SPI-CY): practicability and discriminative validity. Schizophr Res. 2013;146(1–3):69–78. doi:10.1016/J.SCHRES.2013.02.014 23473813

[28] Solmi M, Radua J, Olivola M, Croce E, Soardo L, de Pablo GS, et al. Age at onset of mental disorders worldwide: large-scale meta-analysis of 192 epidemiological studies. Mol Psychiatry. 2021;17:1–15. doi:10.1038/s41380-021-01161-7 PMC8960395 34079068

[29] Fusar-Poli P, Correll CU, Arango C, Berk M, Patel V, Ioannidis JPA. Preventive psychiatry: a blueprint for improving the mental health of young people. World Psychiatry. 2021;20(2):200. doi:10.1002/WPS.20869 34002494 PMC8129854

[30] Fusar-Poli P, Schultze-Lutter F, Cappucciati M, Rutigliano G, Bonoldi I, Stahl D, et al. The dark side of the moon: meta-analytical impact of recruitment strategies on risk enrichment in the clinical high risk state for psychosis. Schizophr Bull. 2016;42(3):732–43. doi:10.1093/SCHBUL/SBV162 26591006 PMC4838090

[31] Fusar-Poli P, Sullivan SA, Shah JL, Uhlhaas PJ. Improving the detection of individuals at clinical risk for psychosis in the community, primary and secondary care: an integrated evidence-based approach. Front Psychiatry. 2019;10:774. doi:10.3389/FPSYT.2019.00774 31708822 PMC6822017

[32] Spada G, Molteni S, Pistone C, Chiappedi M, McGuire P, Fusar-Poli P, et al. Identifying children and adolescents at ultra high risk of psychosis in Italian neuropsychiatry services: a feasibility study. Eur Child Adolesc Psychiatry. 2015;25(1):91–106. doi:10.1007/S00787-015-0710-8 25925786

[33] Molteni S, Filosi E, Mensi MM, Spada G, Zandrini C, Ferro F, et al. Predictors of outcomes in adolescents with clinical high risk for psychosis, other psychiatric symptoms, and psychosis: a longitudinal protocol study. Front Psychiatry. 2019;10:787. doi:10.3389/FPSYT.2019.00787 31849719 PMC6902080

[34] de Pablo GS, De Micheli A, Nieman DH, Correll CU, Kessing LV, Pfennig A, et al. Universal and selective interventions to promote good mental health in young people: systematic review and meta-analysis. Eur Neuropsychopharmacol. 2020;41:28–39. doi:10.1016/J.EURONEURO.2020.10.007 33162291

[35] Fusar-Poli P, Tantardini M, de Simone S, Ramella-Cravaro V, Oliver D, Kingdon J, et al. Deconstructing vulnerability for psychosis: meta-analysis of environmental risk factors for psychosis in subjects at ultra high-risk. Eur Psychiatry. 2017;40:65–75. doi:10.1016/j.eurpsy.2016.09.003 27992836

[36] Raballo A, Poletti M, Preti A, Parnas J. The self in the spectrum: a meta-analysis of the evidence linking basic self-disorders and schizophrenia. Schizophr Bull. 2021;47(4):1007–17. doi:10.1093/SCHBUL/SBAA201 33479736 PMC8266610

[37] Mensi MM, Molteni S, Iorio M, Filosi E, Ballante E, Balottin U, et al. Prognostic accuracy of DSM-5 attenuated psychosis symptoms in adolescents: prospective real-world 5-year cohort study. Schizophr Bull. Published online 3 May 2021. doi:10.1093/SCHBUL/SBAB041 PMC8530398 33939829

[38] Tognin S, van Hell HH, Merritt K, Winter-van Rossum I, Bossong MG, Kempton MJ, et al. Towards precision medicine in psychosis: benefits and challenges of multimodal multicenter studies—PSYSCAN: translating neuroimaging findings from research into clinical practice. Schizophr Bull. 2020;46(2):432–41. doi:10.1093/SCHBUL/SBZ067 31424555 PMC7043057

[39] Fusar-Poli P, de Pablo GS, De Micheli A, Nieman DH, Correll CU, Kessing LV, et al. What is good mental health? A scoping review. Eur Neuropsychopharmacol. 2020;31:33–46. doi:10.1016/J.EURONEURO.2019.12.105 31901337

[40] Fusar-Poli P, Bauer M, Borgwardt S, Bechdolf A, Correll CU, Do KQ, et al. European college of neuropsychopharmacology network on the prevention of mental disorders and mental health promotion (ECNP PMD-MHP). Eur Neuropsychopharmacol. 2019;29(12):1301–11. doi:10.1016/J.EURONEURO.2019.09.006 31606303

